# Discovery of a Novel Insecticidal Peptide with a Cystine-Stabilized α-Helix/α-Helix Motif from the Venom of Scorpion *Liocheles australasiae*

**DOI:** 10.3390/molecules30010032

**Published:** 2024-12-25

**Authors:** Masahiro Miyashita, Naoya Mitani, Fuki Iwamoto, Mitsuki Hirota, Yoshiaki Nakagawa

**Affiliations:** Graduate School of Agriculture, Kyoto University, Kyoto 606-8502, Japan

**Keywords:** scorpion venom, peptide, disulfide, α-helix, κ-KTx, insecticidal, *Liocheles australasiae*

## Abstract

Scorpion venom contains various bioactive peptides, many of which exhibit insecticidal activity. The majority of these peptides have a cystine-stabilized α-helix/β-sheet (CSαβ) motif. In addition to these peptides, scorpion venom also contains those with a cystine-stabilized α-helix/α-helix (CSαα) motif, which are known as κ-KTx peptides. Some of these peptides show weak inhibitory activity on mammal potassium channels, but, in many cases, their biological activity remained unknown. In this study, with the aim of discovering novel insecticidal peptides, we synthesized five peptides, which were predicted to adopt a CSαα motif, identified from the venom of the scorpion *Liocheles australasiae*, and measured their insecticidal activity. As a result, one of the peptides, named LaIT5, exhibited significant insecticidal activity. To the best of our knowledge, this is the first report of insecticidal peptides with a CSαα motif. Furthermore, we synthesized its analogs based on sequence comparisons with other inactive CSαα-motif peptides to identify amino acid residues important for its insecticidal activity. The results indicate that two consecutive His residues at the central region of LaIT5 are particularly important for the activity. Since LaIT5 did not show any toxicity against mice, it was concluded that its action is selective for insects.

## 1. Introduction

Scorpions use venom to capture prey and to defend against predators. The venom contains a variety of components such as peptides, proteins, nucleic acids, lipids, and inorganic salts [1,2,3]. Among them, peptides are the major bioactive components, and many of them show toxicity to insects and/or mammals [4]. In addition to these, some of the venom components are known to exhibit antibacterial, antiviral, and analgesic effects, although their biological significance in the venom remains unclear [5,6]. Therefore, scorpion venom is considered to be an attractive source of bioactive peptides, which can be applied to agrochemicals and pharmaceuticals [7,8].

Based on their structural characteristics, scorpion venom peptides are classified into two groups: non-disulfide bridged peptides (NDBPs) and disulfide bridged peptides (DBPs). NDBPs have been mostly identified as an antimicrobial peptide [9]. They form pores in the membranes of microbes due to their amphiphilic α-helical structure. Thus, NDBPs probably play a role in preventing the pathogen infection through the venom gland. The effect of NDBPs on biological membranes is also observed against insect cells to exhibit insecticidal activity in some cases [10]. On the other hand, DBPs, which form unique structural motifs through the cross-linking of multiple disulfide bonds, are the main components responsible for the toxicity of scorpion venom [7]. They mainly act on ion channels (Na^+^, K^+^, Cl^−^, Ca^2+^) as neurotoxins, causing paralysis, pain, and eventually death in the target organism [7,11]. In many cases, the action of DBPs on target molecules is highly specific, and this property meets the requirements for agrochemicals and pharmaceuticals [8,12]. Many DBPs in scorpion venom are known to have a cystine-stabilized α-helix/β-sheet (CSαβ) motif, which contains 3–4 disulfide bridges [11]. In addition to these peptides, there are also peptides with various structural motifs, such as an inhibitor cystine knot (ICK) motif, disulfide-directed hairpin (DDH) motif, and a cystine-stabilized α-helix/α-helix (CSαα) motif, which contains 2–3 disulfide bridges, in scorpion venom [11]. To date, many peptides with a CSαβ motif or a DDH motif have been shown to be insecticidal, but there are no reports of insecticidal peptides with an ICK or a CSαα motif from scorpion venom [8].

We have been investigating the bioactive peptides in the venom of scorpion *Liocheles australasiae* (Hormuridae family) and identified several insecticidal peptides with a CSαβ or a DDH motif [13,14,15]. However, given that more than 200 components are present in *L. australasiae* venom, peptides with structural motifs other than CSαβ and DDH motifs may exhibit insecticidal activity. In this study, with the aim of discovering novel insecticidal peptides, we synthesized peptides that were predicted to adopt a CSαα motif in a previous transcriptome analysis of the venom gland of *L. australasiae* [16] and measured their insecticidal activity. Furthermore, for the peptide that showed insecticidal activity, we synthesized its analogs to identify amino acid residues important for activity.

## 2. Results

### 2.1. Identification of κ-KTx-like Peptides

**Figure 1:**
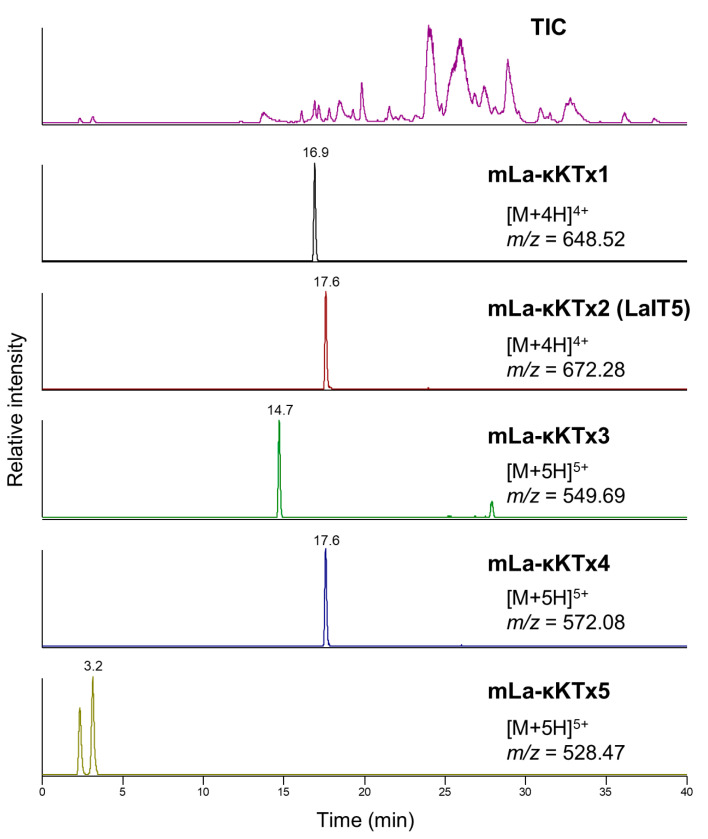
Detection of κ-KTx-like peptides with a predicted mature structure in the *L. australasiae* venom using high-resolution LC/MS analysis.

The precursor sequences of the peptides that are predicted to adopt a CSαα motif, which are also known as a κ-KTx peptide, were obtained previously (Table 1) [16]. After prediction of signal peptides, propeptide regions and C-terminal amidation for each precursor, the presence of putative mature peptides (mLa-κKTx1~5) in the venom was examined using high-resolution LC/MS analysis. As a result, all of the peptides with molecular masses calculated from each putative mature structure were observed (Table 2 and Figure 1). Furthermore, the HPLC fractions containing each peptide were enzymatically digested and subjected to LC/MS analysis. Molecular masses of digested fragments observed were consistent with those calculated from the putative sequences of each peptide (Figure S1). These results clearly indicate that these κ-KTx-like peptides are present in the *L. australasiae* venom.

The κ-KTx-like peptides observed were synthesized by the Fmoc solid-phase method. Formation of disulfide bridges was carried out using redox buffer containing oxidized and reduced glutathione (Figure S2). To confirm that the synthesized peptides are identical to the peptides in the venom, retention times in the LC/MS analysis were compared between them. As a result, both peptides eluted at the same retention time, indicating that they are identical (Figure S3). Furthermore, each synthetic peptide was enzymatically digested without reducing the disulfide bonds and analyzed by LC/MS. Based on the observed molecular masses of the digested fragments, it was confirmed that all peptides have the disulfide bonding pattern of the CSαα motif (Table 2 and Figure S4).

The 3D structures of these peptides were predicted using AlphaFold3 (Figure S5). All peptides except mLa-κKTx3 were predicted to adopt a CSαα motif. Unexpectedly, mLa-κKTx3 was predicted to be composed of two β-sheets rather than two α-helices, although the disulfide bonding pattern is the same as in the CSαα motif. This is likely because the loop region between the second and third Cys residues of mLa-κKTx3 is shorter than that of other peptides. In this study, although this peptide may not adopt a CSαα motif, its insecticidal activity was evaluated.

### 2.2. Biological Activity

The insecticidal activity of the synthesized κ-KTx-like peptides was measured against crickets (*Acheta domesticus*). Since this scorpion preys on crickets in its natural environment, it was expected that the venom would contain various peptides showing insecticidal activity against crickets. As a result, it was found that the mLa-κKTx2 showed significant insecticidal activity, whereas no activity was observed for other peptides, even at a dose of 80 nmol/g (Table 3). Here, we renamed mLa-κKTx2 to LaIT5 based on its activity and the number of the identified insecticidal toxins in the *L. australasiae* venom. The LD_50_ value of LaIT5 was determined to be 10 nmol/g. Crickets injected with LaIT5 showed symptoms of leg cramps, followed by strong abdominal contractions. This suggests that LaIT5 acts on the muscle tissue of insects. On the other hand, when LaIT5 was injected intracerebroventricularly or intraperitoneally into mice at a dose of 0.2 or 5 µg/g of body weight, respectively, no toxic symptoms were observed.

### 2.3. Structure–Activity Relationship

**Figure 2:**
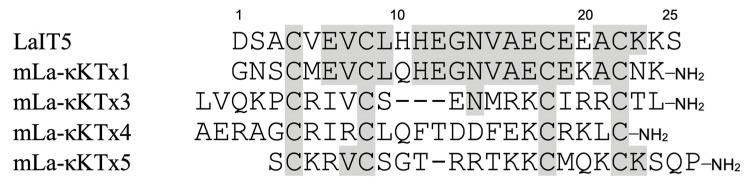
Comparison of the sequences between LaIT5 and other κ-KTx-like peptides. Identical residues between LaIT5 and other peptides were shaded.

mLa-κKTx1 showed no insecticidal activity despite its high sequence homology with LaIT5 (about 70%, Figure 2). Therefore, we thought that some of the amino acid residues different between mLa-κKTx1 and LaIT5 would be critical for the insecticidal activity. First, we focused on the 10th His and 20th Glu residues of LaIT5. This is because these residues have different properties from the corresponding residues in mLa-κKTx1: basic (His) vs. neutral (Gln), and acidic (Glu) vs. basic (Lys). Therefore, we synthesized analogs **1** and **2**, in which the His or Glu residue in LaIT5 was substituted with Gln or Lys, respectively (Table 4). Second, the C-terminal carboxyl group of LaIT5 was converted to an amide group, as observed in mLa-κKTx1 (analog **3**). As a result, the insecticidal activity of analog **1** significantly decreased (Table 4), but analogs **2** and **3** were equipotent to the original LaIT5. This suggests that the 10th His residue is critical for the insecticidal activity of LaIT5. To confirm the importance of the 10th His residue in LaIT5, we synthesized analog **4**, in which the corresponding Gln residue of mLa-κKTx1 was substituted with His. As expected, this analog showed insecticidal activity comparable to that of LaIT5, confirming the importance of the 10th His residue.

We then investigated the importance of other residues in the common sequence between LaIT5 and mLa-κKTx1. Acidic (Asp and Glu) or basic residues (Lys and His) common to LaIT5 and mLa-κKTx1 were substituted with Ala (analogs **5**–**13**) and tested for insecticidal activity (Table 4). The substitution of the N-terminal Asp (analog **5**) had little effect on activity, whereas substitution of the sixth Glu residue (analog **6**) decreased activity by a factor of two. This indicates that the sixth Glu residue also partially contributes to the activity. When the 11th His residue was substituted with Ala (analog **7**), the activity greatly decreased by a factor of five. This suggests that the presence of two consecutive His residues at the 10th and 11th positions in LaIT5 is particularly important for the activity. The substitution of the 12th Glu (analog **8**) did not affect the activity of LaIT5. Interestingly, analogs **9**–**13**, in which the Glu and Lys residues in the C-terminal half region were substituted with Ala, showed 2–3 times higher activity than the original LaIT5.

## 3. Discussion

In this study, we discovered a novel insecticidal peptide LaIT5 from the κ-KTx-like peptides present in *L. australasiae* venom. To the best of our knowledge, this is the first report of an insecticidal peptide with a CSαα motif from scorpion venom. The LD_50_ value of LaIT5 (10 nmol/g) was between that of LaIT1 (5.2 nmol/g) and LaIT3 (12 nmol/g), the insecticidal peptides identified from the venom of *L. australasiae* [13,15]. This indicates that LaIT5 is moderately potent in this venom. Since this peptide did not show any toxic symptoms after administration to mice, it was concluded that the action of LaIT5 is highly selective against insects.

**Figure 3:**
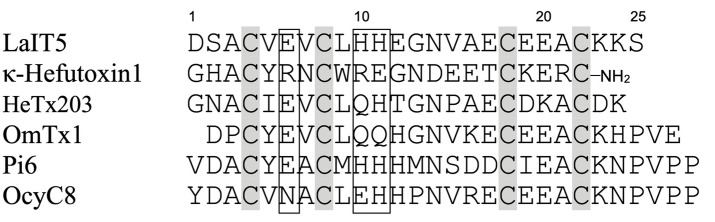
Multiple sequence alignment of peptides showing similarity to LaIT5. Cys residues are shaded and the regions important for insecticidal activity of LaIT5 are boxed.

The results of the structure–activity relationship study of LaIT5 showed that the two consecutive His residues at the 10th and 11th positions are particularly important for insecticidal activity, and that the Glu at the 6th position is also partially involved in the activity. When the sequences of the κ-KTx-like peptides in the *L. australasiae* venom are compared, only LaIT5 contains these three residues (Figure 2). This clearly explains the reason why no insecticidal activity was observed in the κ-KTx-like peptides other than LaIT5. The sequence of LaIT5 was further compared with that of similar peptides from other scorpion species (Figure 3). Of these, Pi6 contains not only two consecutive His residues at the central region, but also the 6th Glu residue, suggesting that it may have insecticidal activity. The peptides other than Pi6 are unlikely to exhibit insecticidal activity due to the absence of these two His residues. Pi6 was shown to inhibit mammal K^+^ channels, but it requires relatively high concentrations [17]. Therefore, it is not surprising to assume that the target of this peptide is insects rather than mammals, and its synthesis and activity evaluation is currently underway. In the near future, it is expected that other CSαα-motif peptides that exhibit insecticidal activity will be found in the venom of various scorpion species.

**Figure 4:**
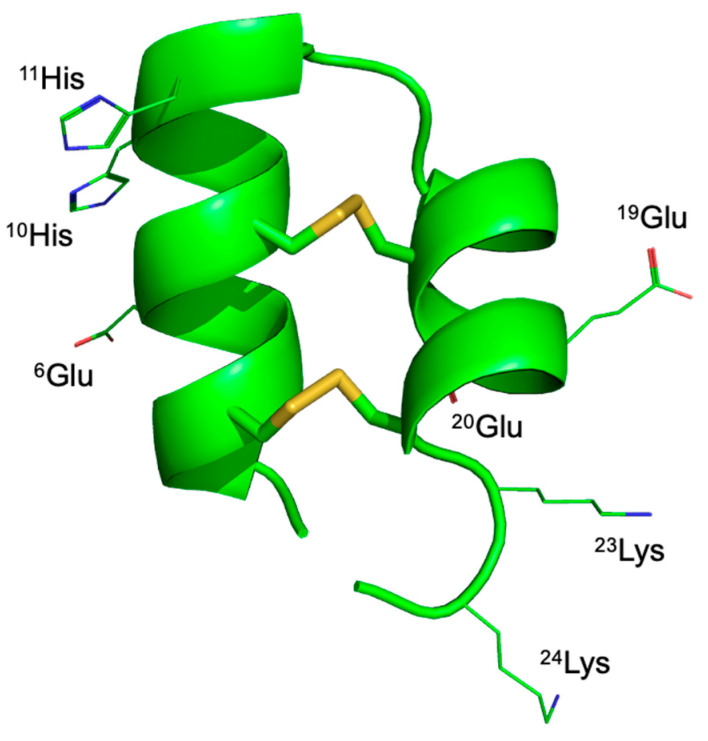
Predicted 3D structure of LaIT5.

In the 3D structure of LaIT5 predicted by AlphaFold3 (Figure 4), two consecutive His residues critical for the activity are located near the central loop region on the N-terminal α-helix. Although the action target of LaIT5 is unknown, these His residues may be involved in interactions with its target molecule. However, Glu and Lys residues, whose substitution caused an increase in activity, are located on the C-terminal α-helix. Since the increase in activity was observed by replacing either a Glu or Lys residue with Ala at several positions, it is unlikely that the basicity or acidity of the side chain structure at a specific position is important. Rather, an increase in hydrophobicity induced by the substitution of these residues may affect the overall structure of the peptide or its distribution within the insect body.

There have been many reports on peptides with a structure similar to that of those acting on K^+^ channels (KTx peptides) in scorpion venom [18]. KTx peptides are classified into seven families (α, β, ɤ, κ, δ, λ, and ε) according to their structures and actions. Of the seven families of KTx peptides, the α-KTx peptides, which have a CSαβ motif, have been the most extensively studied, with nearly 300 sequences in the database. The β-KTx peptides also contain a CSαβ motif but have an additional linear α-helix domain on their N-terminal side. It is known that some α- and β-KTx peptides contribute to the insecticidal activity of scorpion venom [13,14,19,20]. However, no insecticidal peptides have been found from peptides belonging to families other than the α- and β-KTx families. In *L. australasiae* venom, the presence of KTx peptides belonging to the α-, β-, κ-, and δ-families was predicted by transcriptome analysis [16]. Of these, two types of β-KTx peptides were shown to exhibit insecticidal and antibacterial activities [13,14], but the functions of other KTx family peptides remained unknown. In this study, we found LaIT5 as an insecticidal peptide from CSαα-motif peptides, which structurally belong to the κ-KTx family, suggesting that these peptides also contribute to the insecticidal activity of scorpion venom.

Since LaIT5 has a structure similar to that of κ-KTx peptides, it may act on K^+^ channels. However, we cannot rule out the possibility that it acts on other target molecules. By evaluating its interaction with the possible receptors (ion channels) in silico, it is possible to find candidate target molecules of LaIT5, which will be further proven through electrophysiological experiments.

**Figure 5:**
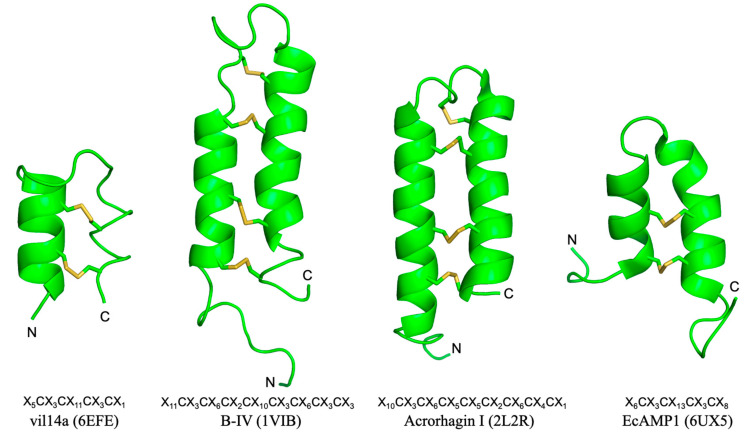
Three-dimensional structure and position of Cys residues of peptides with a CSαα motif. Characters in parentheses indicate PDB IDs.

Peptides with a structure similar to the scorpion CSαα motif have been found in various organisms (Figure 5). Conotoxin vil14a (PDB: 6EFE) identified from cone snail (*Conus villepinii*) venom has a structure with two α-helices cross-linked by two disulfide bonds, as observed in CSαα-motif peptides [21]. Although this peptide has inhibitory activity against mammal K^+^ channels, its effect was very weak as observed for scorpion κ-KTx peptides, suggesting that the peptide has other unknown functions. B-IV (PDB: 1VIB) found in marine worms (*Cerebratulus lacteus*) has two α-helices, but its sequence is longer than that of scorpion κ-KTx peptides and cross-linked by four disulfide bonds [22]. This peptide has toxicity to crustaceans by acting on their Na^+^ channels. Acrorhagin I (PDB: 2L2R), identified in sea anemones (*Actinia equina*), also has two α-helices cross-linked by four disulfide bonds, one of which is cross-linked between the α-helix and the loop [23]. This peptide is also toxic to crustaceans, but, unlike B-IV, its toxicity is suggested to be due to the chelation of metal ions via multiple His residues [24]. In this regard, there could be a similarity to the fact that two His residues are important for the activity of LaIT5. However, since LaIT5 retains activity even after the substitution of one of the His residues with Ala, it is unlikely that LaIT5 has a similar mechanism of action to Acrorhagin I. Peptides with a CSαα motif have also been found in plants, which are known as α-hairpinins [25]. MBP-1 identified from maize (*Zea mays*) is the first member of α-hairpinin [26]. Since most of the α-hairpinins, including MBP-1, show antimicrobial activity, these are thought to contribute to innate immunity in plants. The mechanism of action of α-hairpinins remains poorly understood, but it is suggested that intracellular action, rather than membrane disruption, is involved. The 3D structure of α-hairpinins has been reported for EcAMP1 (PDB: 6UX5) from barnyard grass (*Echinochloa crus-galli*), which has a high sequence similarity to MBP-1 [27]. Thus, peptides with a structure similar to the scorpion κ-KTx have been discovered in a wide variety of organisms, but their sequence lengths, positions of disulfide bonds, and biological activities are diverse (Figure 5). This implies that the CSαα motif is important as a scaffold structure for bioactive peptides, and that each organism acquired peptides with the CSαα motif to exert various biological functions as a result of convergent evolution. Although no CSαα-motif peptides other than LaIT5 have been found to exhibit insecticidal activity so far, it is expected that they will be discovered in organisms other than scorpions in the near future. Furthermore, these peptides are promising as lead compounds for the development of biopesticides.

## 4. Materials and Methods

### 4.1. Materials

The venom of *L. australasiae* was collected in our laboratory as previously described [15]. 1-[Bis(dimethylamino)methylene]-1H-1,2,3-triazolo[4,5-b]pyridinium 3-oxide hexafluorophosphate (HATU) was purchased from Tokyo Chemical Industry (Tokyo, Japan). 1-Hydroxybenzotriazole (HOBt) was purchased from Watanabe Chemical Industries (Hiroshima, Japan). Rink amide AM resin (0.70 mmol/g) was obtained from Novabiochem (Billerica, MA, USA). Rink amide ProTide (LL) resin, Fmoc-Ser(tBu)-Wang-ProTide (LL) resin, and Oxyma Pure were purchased from CEM Corporation (Charlotte, NC, USA). *N*,*N*’-diisopropylcarbodiimide (DIC) and lysyl endopeptidase (mass spectrometry grade), endoproteinase Glu-C (sequencing grade), and trypsin (mass spectrometry grade) were obtained from FUJIFILM Wako Pure Chemical Corporation (Osaka, Japan). All other chemicals were commercially available.

### 4.2. Venom Fractionation

The lyophilized venom was dissolved in 2% acetic acid and separated by reversed-phase HPLC using a C4 column (Vydac PROTEIN C4, 10 × 250 mm, Hichrom, Reading, UK). The column was eluted with 0.1% TFA/water and 0.1% TFA/acetonitrile at a flow rate of 2 mL/min using a 60 min linear gradient from 5–60% of acetonitrile at 40 °C. Elution was monitored by absorption at 215 nm.

### 4.3. Mass Spectrometry

For analysis of peptides in the venom, an Orbitrap Exploris 240 mass spectrometer (Thermo Fisher Scientific, Waltham, MA, USA) equipped with a heated-ESI source in the positive mode at 60,000 resolution was used. HPLC separation was carried out on a C18 column (Aeris PEPTIDE XB-C18, 2.6 μm, 2.1 × 150 mm, Phenomenex, Torrance, CA, USA); this column was eluted with 0.1% formic acid/water and 0.1% formic acid/acetonitrile at a flow rate of 0.3 mL/min using a 60 min linear gradient of 5–50% of acetonitrile. For analysis of synthesized peptides and their disulfide bonding patterns, an LCMS-IT-TOF mass spectrometer (Shimadzu), an LCMS-8030 mass spectrometer (Shimadzu), or an LCMS-2020 mass spectrometer (Shimadzu) equipped with an electrospray ion source in the positive mode were used. HPLC separation was carried out on a C18 column (TSKgel ODS-100V, 3 μm, 1.0 × 150 mm, Tosoh, Tokyo, Japan); this column was eluted with 0.1% formic acid/water and 0.1% formic acid/acetonitrile at a flow rate of 50 µL/min using a 35 min linear gradient of 2–40% acetonitrile; or a C18 column (TSKgel ODS-100V, 3 μm, 2.0 × 150 mm, Tosoh) was eluted with 0.1% formic acid/water and 0.1% formic acid/acetonitrile at a flow rate of 0.2 mL/min using a 30 min linear gradient of 10–40% acetonitrile; or a C18 column (Aeris PEPTIDE XB-C18, 2.6 μm, 2.1 × 150 mm, Phenomenex) was eluted with 0.1% formic acid/water and 0.1% formic acid/acetonitrile at a flow rate of 0.2 mL/min using a 45 min linear gradient of 5–50% of acetonitrile.

### 4.4. Sequence Analysis

Signal peptides were predicted using SignalP (Ver. 5.0) [28] for precursor sequences obtained by transcriptome analysis. Propeptide regions were predicted using ProP (ver. 1.0) [29] or by comparing the sequences with those of similar peptides. When the C-terminus of the precursor sequence contained Gly or Gly-Arg, it was assumed that the C-terminus was amidated. Multiple sequence alignment was conducted using Clustal Omega [30]. The sequences of peptides with a CSαα motif derived from other organisms were obtained manually from public databases based on the descriptions in the literature.

### 4.5. Peptide Synthesis

Peptides were synthesized manually or via an automated synthesizer (Liberty Light, CEM, Charlotte, NC, USA) using the Fmoc-based SPPS method. For the synthesis of mLa-κKTx1, mLa-κKTx3, mLa-κKTx4, and mLa-κKTx5, Rink amide AM resin was used. For the synthesis of LaIT5 and its analogs, Fmoc-Ser(tBu)-Wang-ProTide (LL) was used. For the synthesis of an mLa-κKTx1 analog and a C-terminal amidated LaIT5 analog, Rink amide ProTide (LL) resin was used. In the manual synthesis, Fmoc deprotection was performed with 20% piperidine in *N,N*-dimethylformamide (DMF) twice under microwave irradiation (Initiator+, Biotage, Uppsala, Sweden) at 80 °C, each for 0.5 and 3 min. Each Fmoc-protected amino acid (3 eq) was coupled in the presence of HATU (3 eq) and *N,N*-diisopropylethylamine (DIEA, 6 eq) in DMF under microwave irradiation at 75 °C for 5 min. For the introduction of Cys, DIC (3 eq) and HOBt (3 eq) were used instead of the reagents shown above, and the mixture was reacted under microwave irradiation at 50 °C for 20 min. When using the automated synthesizer, Fmoc deprotection was performed with 20% piperidine in DMF at 75 °C for 15 s and at 90 °C for 50 s under microwave irradiation. Each Fmoc-protected amino acid (5 eq) was coupled in the presence of Oxyma Pure (5 eq), DIC (10 eq) and DIEA (0.5 eq) at 75 °C for 15 s and at 90 °C for 110 s under microwave irradiation. For introduction of His, the mixture was reacted at 25 °C for 120 s and at 50 °C for 480 s under microwave irradiation. After the completion of coupling reaction, the resin was sequentially washed with DMF, diethyl ether and methanol and dried in vacuo. The cleavage of peptides from the resin and removal of side-chain protecting groups were carried out using a TFA solution containing 2.5% H_2_O, 2.5% 1,2-ethandithiol and 1.0% triisopropylsilane as scavengers. After the resin was removed by filtration, cold diethyl ether was added to the filtrate to precipitate the peptides, followed by washing with cold diethyl ether twice and drying in vacuo.

### 4.6. Folding Reaction

Disulfide bond formation was performed in a redox buffer. Linear peptides were dissolved at a concentration of 0.1 mg/mL (1.0 mg/mL for LaIT5 analogs) in 200 mM Tris-HCl (pH 8.0, adjusted with HCl) buffer containing 1 mM reduced glutathione and 0.1 mM oxidized glutathione. The mixture was incubated overnight at 25 °C, and the progress of the folding reaction was monitored using LC/MS. The correctly folded peptide was subsequently purified using HPLC and lyophilized. Preparative HPLC was carried out using a C18 column (Inertsil ODS-3, 20 × 250 mm, GL Sciences, Tokyo, Japan). The column was eluted with 0.1% TFA/water and 0.1% TFA/acetonitrile at a flow rate of 7 mL/min using a 25 min linear gradient from 10 to 40% of acetonitrile at 40 °C. Elution was monitored by absorption at 215 nm. The results of the LC/MS analysis of purified peptides are shown in Figures S2 and S6.

### 4.7. Determination of Disulfide Bonding Patterns

Each synthetic peptide (100 pmol) was dissolved in 50 mM Tris-HCl (pH 8.0) buffer and mixed with Glu-C (0.1 µg for mLa-κKTx1 and LaIT5), trypsin (0.1 µg for mLa-κKTx3 and mLa-κKTx4), or Lys-C (0.1 µg for mLa-κKTx5) at 37 °C. After incubation for 18 h, the digested peptides were subjected to LC/MS analysis.

### 4.8. 3D Structure Prediction

Three-dimensional structures of the peptides were modeled using AlphaFold3 [31]. The 3D structure of each peptide was visualized using PyMol (Ver. 3.0, Schrödinger, LLC, New York, NY, USA).

### 4.9. Bioassay

The insecticidal activity of synthetic peptides was tested using crickets (*Acheta domesticus*) as this scorpion species preys on crickets in its natural environment. Each peptide was dissolved in distilled water and injected into the abdominal region of the specimens (50 ± 5 mg of body weight). Distilled water was used as a control. Ten crickets were used for each measurement, and the number of dead crickets was counted 48 h after injection. The dose required to induce death in half the number of crickets (LD_50_) was determined using PRISM statistical software (Ver. 4.0, GraphPad Software, La Jolla, CA, USA). Toxicity to mammals was evaluated by injecting the sample solution in a PBS buffer intracerebroventricularly or intraperitoneally into mice (male Slc:ICR strain, 20 g of body weight) after anesthetizing with isoflurane. The PBS buffer was injected as a negative control. Three animals were used for each measurement, and the toxic symptoms were monitored for up to 24 h. The experimental protocol was approved by the Ethical Committee for the Welfare of Animals at Kyoto University.

## Figures and Tables

**Table 1:** Prediction of mature sequences of κ-KTx-like peptide in the *L. australasiae* venom.

Precursor	Sequence ^1)^
La-κKTx1	MKPSTSAYALLLVLTFGIITSGVFA *VPMDEENTFEVEKR* **GNSCMEVCLQHEGNVAECEKACNK** *G*
La-κKTx2	MKPSTSAFILLLVLTFGIITSGVSA *IPMDEENTFEEQKR* **DSACVEVCLHHEGNVAECEEACKKS**
La-κKTx3	MKLLPLLVILIICALMANEAFC *DQGARERSENLEDTRD* **LVQKPCRIVCSENMRKCIRRCTL** *GR*
La-κKTx4	MKPSSFAIALILVLFLGFTNA *VSGEYAESISGDRMER* **AERAGCRIRCLQFTDDFEKCRKLC** *G*
La-κKTx5	MKLLPLLLVILIVCALLPNEAFC *DQSAVERSESLEEVSREIVKR* **SCKRVCSGTRRTKKCMQKCKSQP** *GR*

^1)^ Signal sequences are underlined, propeptide regions are shown in italics, mature sequences are shown in bold, and C-terminal amidation sites are double-underlined.

**Table 2:** Determination of disulfide bonding pattern of the κ-KTx-like peptides in the *L. australasiae* venom by LC/MS analysis.

Peptide (Enzyme)	Mature Structure	Molecular Mass		Observed Digest	Molecular Mass	
		Calcd. ^1)^	Obsd.		Calcd. ^1)^	Obsd.
mLa-κKTx1 (Glu-C)	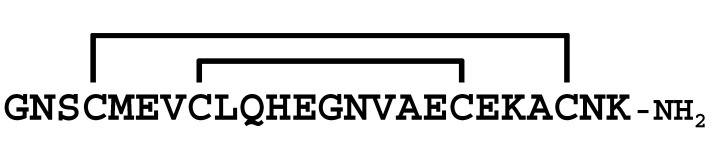	2590.05	2590.05	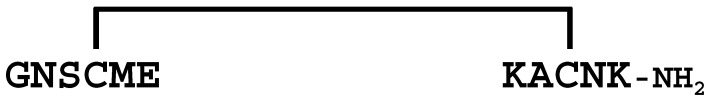	1198.5	1198.4
				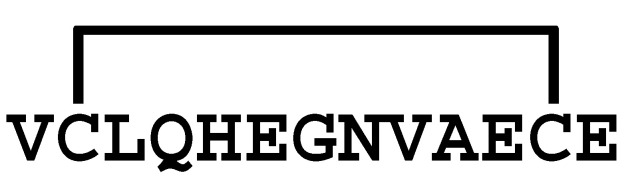	1427.6	1427.6
mLa-κKTx2 (Glu-C)	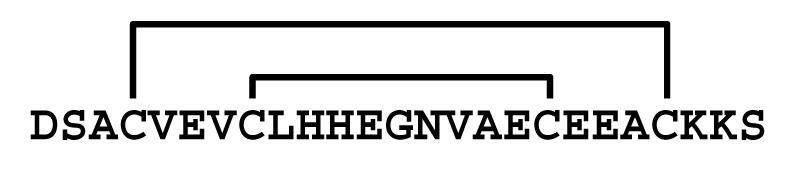	2685.09	2685.09	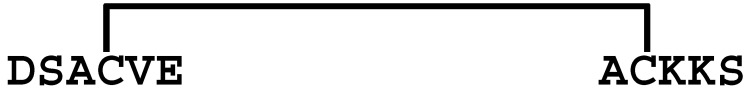	1155.5	1155.4
				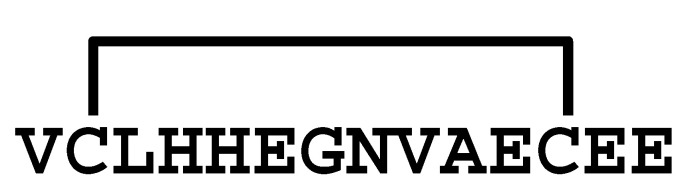	1565.6	1565.6
mLa-κKTx3 (Trypsin)	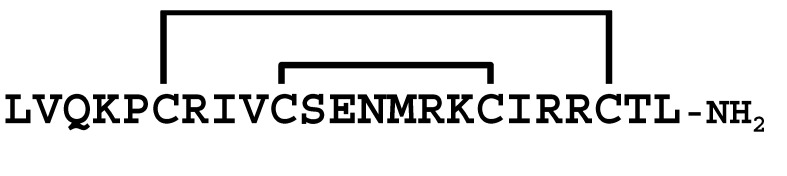	2743.42	2743.41	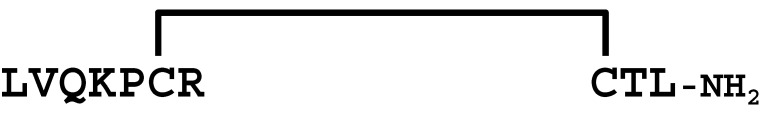	1174.6	1174.6
				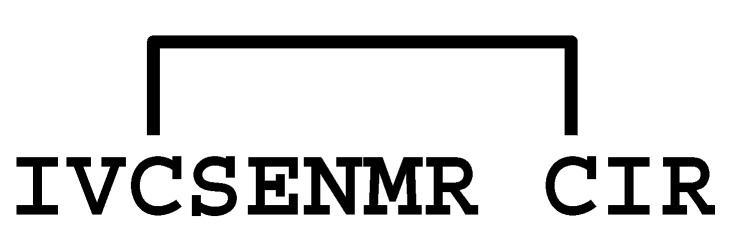	1338.6	1338.6
mLa-κKTx4 (Trypsin)	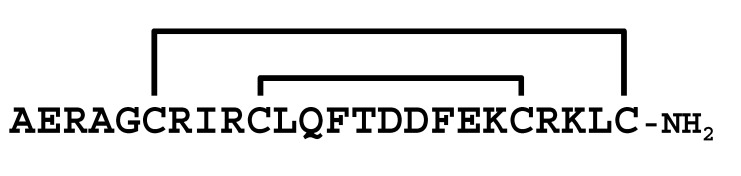	2855.36	2855.35	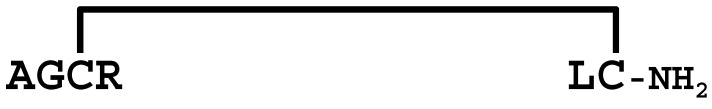	636.3	636.4
				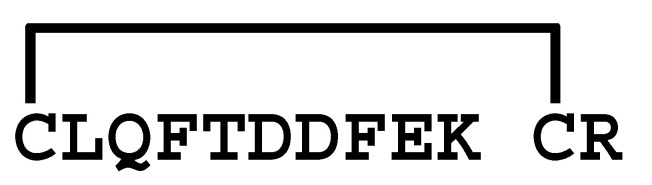	1519.6	1519.7
mLa-κKTx5 (Lys-C)	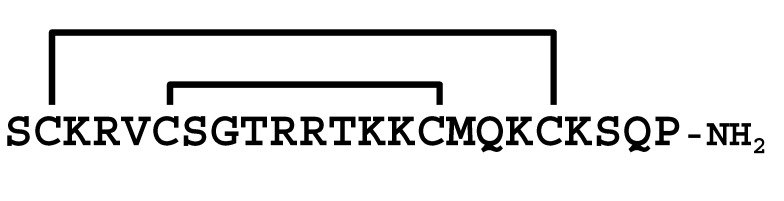	2637.30	2637.30	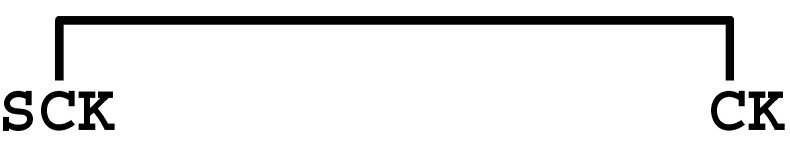	583.2	583.3
				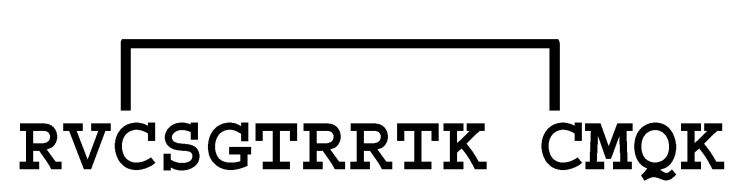	1668.8	1669.2

^1)^ Values were calculated using a structure containing disulfide bridges.

**Table 3:** Insecticidal activity of κ-KTx-like peptides identified in *L. australasiae* venom. Activity was evaluated by injecting each peptide solution into crickets.

Peptide	LD_50_ (nmol/g)
mLa-κKTx1	>80
mLa-κKTx2 (LaIT5)	10
mLa-κKTx3	>80
mLa-κKTx4	>80
mLa-κKTx5	>80

**Table 4:** Insecticidal activity of analogs of LaIT5 and mLa-κKTx1. Activity was evaluated by injecting each peptide solution into crickets.

No.	Analogs	LD_50_ (nmol/g)
	LaIT5	10
**1**	LaIT5(H10Q)	53
**2**	LaIT5(E20K)	12
**3**	LaIT5(NH_2_)	8.7
**4**	mLa-κKTx1(Q10H)	9.0
**5**	LaIT5(D1A)	14
**6**	LaIT5(E6A)	21
**7**	LaIT5(H11A)	45
**8**	LaIT5(E12A)	9.7
**9**	LaIT5(E17A)	4.3
**10**	LaIT5(E19A)	5.5
**11**	LaIT5(E20A)	3.1
**12**	LaIT5(K23A)	5.0
**13**	LaIT5(K24A)	3.4

## Data Availability

The original contributions presented in this study are included in the article/Supplementary Materials. Further inquiries can be directed to the corresponding author.

## Supplementary Materials

The following supporting information can be downloaded at: https://www.mdpi.com/article/10.3390/molecules30010032/s1, Figure S1: LC/MS analysis of digested fragments of κ-KTx-like peptides in the *L. australasiae* venom; Figure S2: LC/MS analysis of the synthesized κ-KTx-like peptides after HPLC purification; Figure S3: Comparison of LC/MS retention times between the synthesized κ-KTx-like peptides and those in the HPLC fraction of the *L. australasiae* venom; Figure S4: Confirmation of disulfide bonding patterns of the synthesized κ-KTx-like peptides by LC/MS analysis of fragments digested without reducing the disulfide bonds; Figure S5: Prediction of 3D structures of the κ-KTx-like peptides in *L. australasiae* venom; Figure S6: LC/MS analysis of the synthesized LaIT5 analogs after HPLC purification.
