# Supplementary material for: Discovery of a Novel Insecticidal Peptide with a Cystine-Stabilized α-Helix/α-Helix Motif from the Venom of Scorpion Liocheles australasiae
Source: Molecules. 2024 Dec 25;30(1):32. doi: 10.3390/molecules30010032 (PMC11722136; doi:10.3390/molecules30010032)
Supplement: Supplementary file 1 [file molecules-30-00032-s001.zip › molecules-3321082-supplementary.pdf]

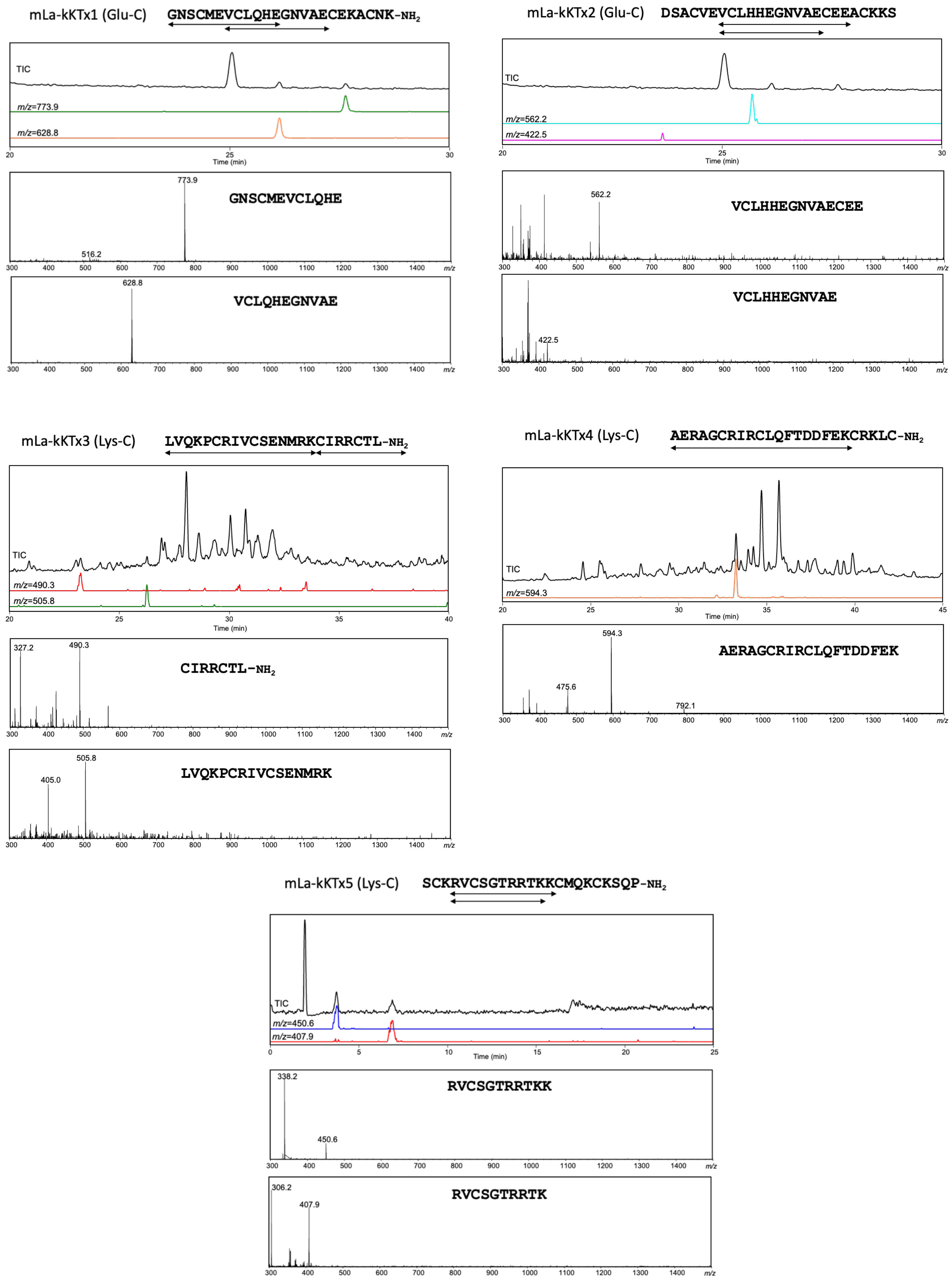

Figure S1 LC/MS analysis of digested fragments of κ-KTx-like peptides in the *L. australasiae* venom.

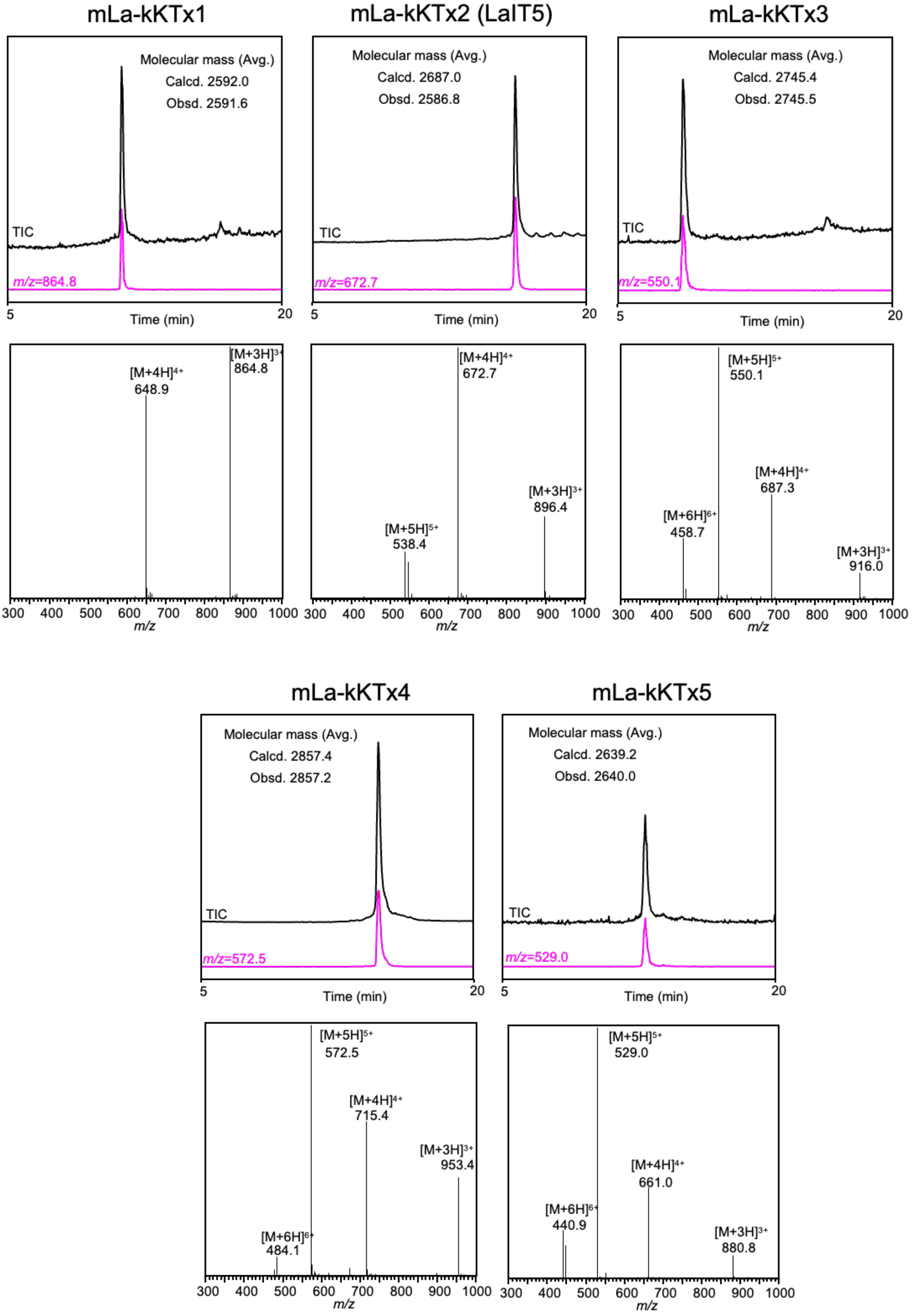

Figure S2 LC/MS analysis of the synthesized κ-KTx-like peptides after HPLC purification.

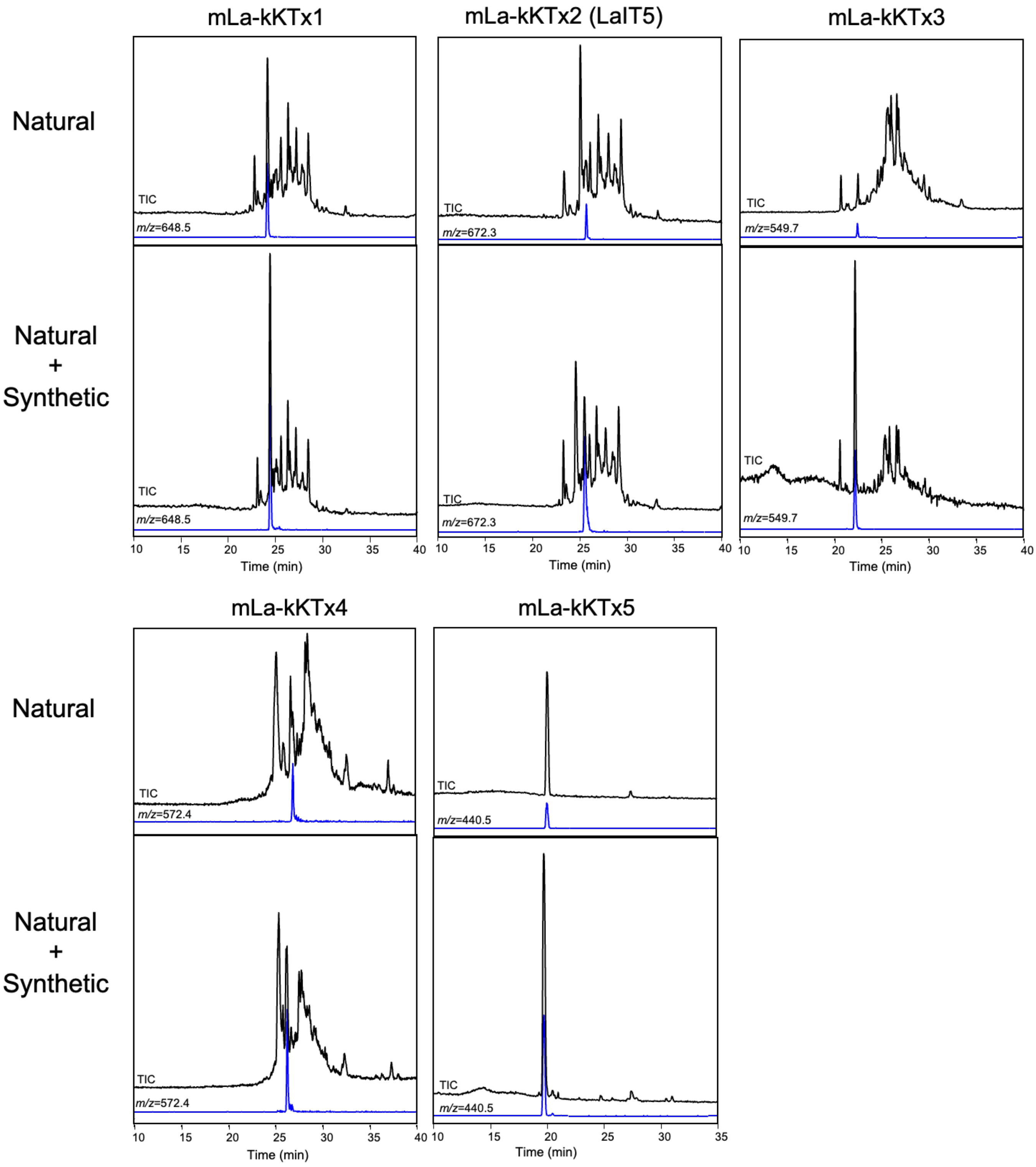

Figure S3 Comparison of LC/MS retention times between the synthesized κ-KTx-like peptides and those in the HPLC fraction of the *L. australasiae* venom.

mLa-κKTx1 after Glu-C digestion

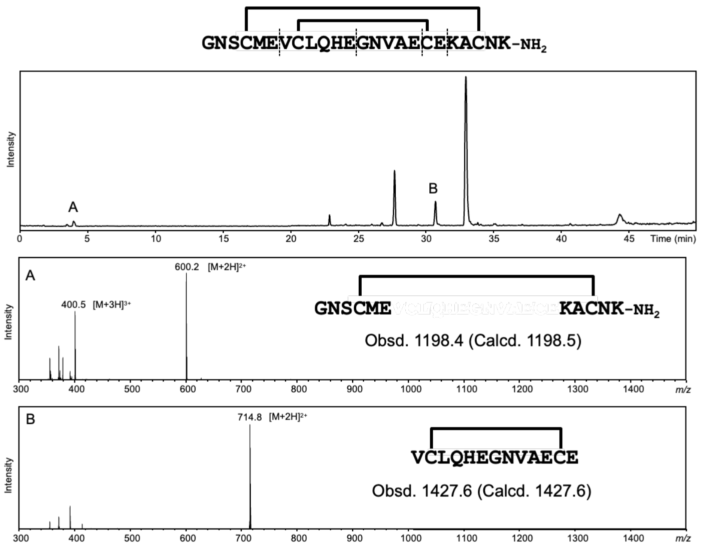

mLa-κKTx2 (LaIT5) after Glu-C digestion

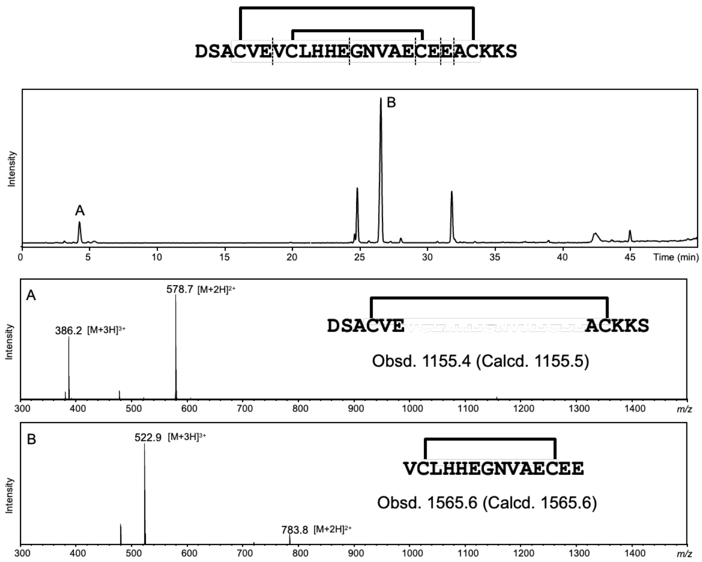

mLa-κKTx3 after trypsin digestion

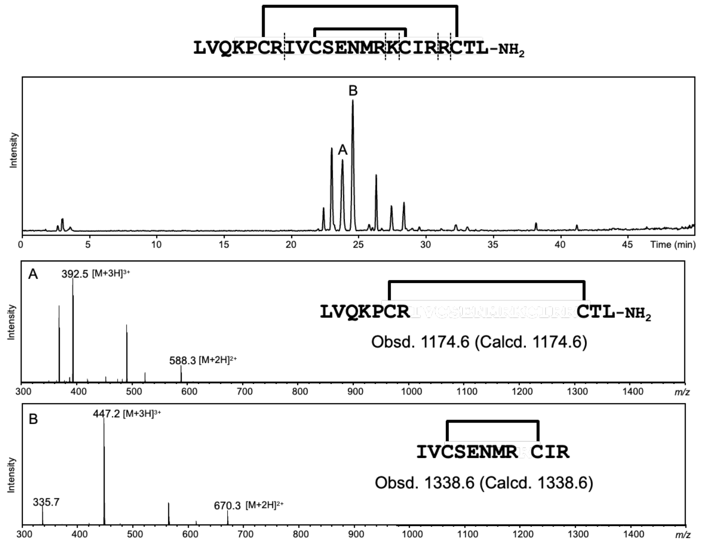

mLa-κKTx4 after trypsin digestion

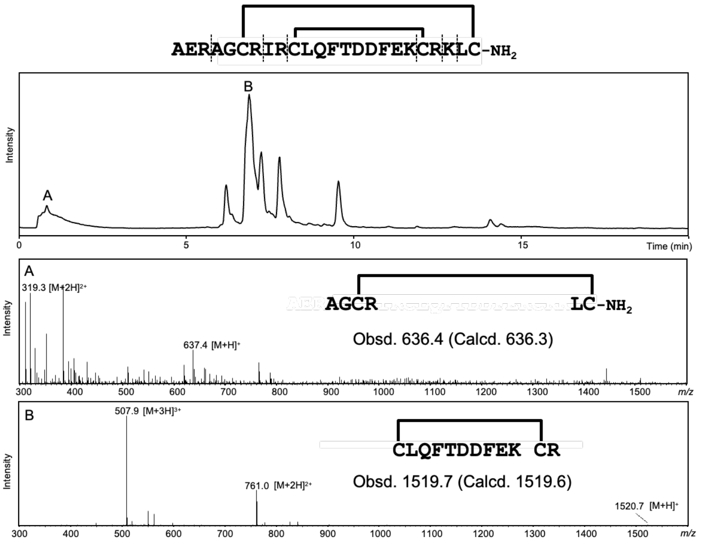

mLa-κKTx5 after Lys-C digestion

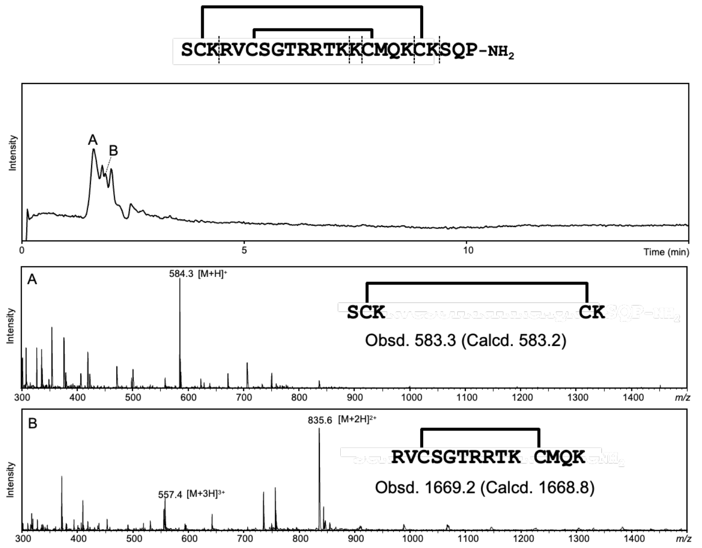

Figure S4 Confirmation of disulfide bonding patterns of the synthesized κ-KTx-like peptides by LC/MS analysis of fragments digested without reducing the disulfide bonds.

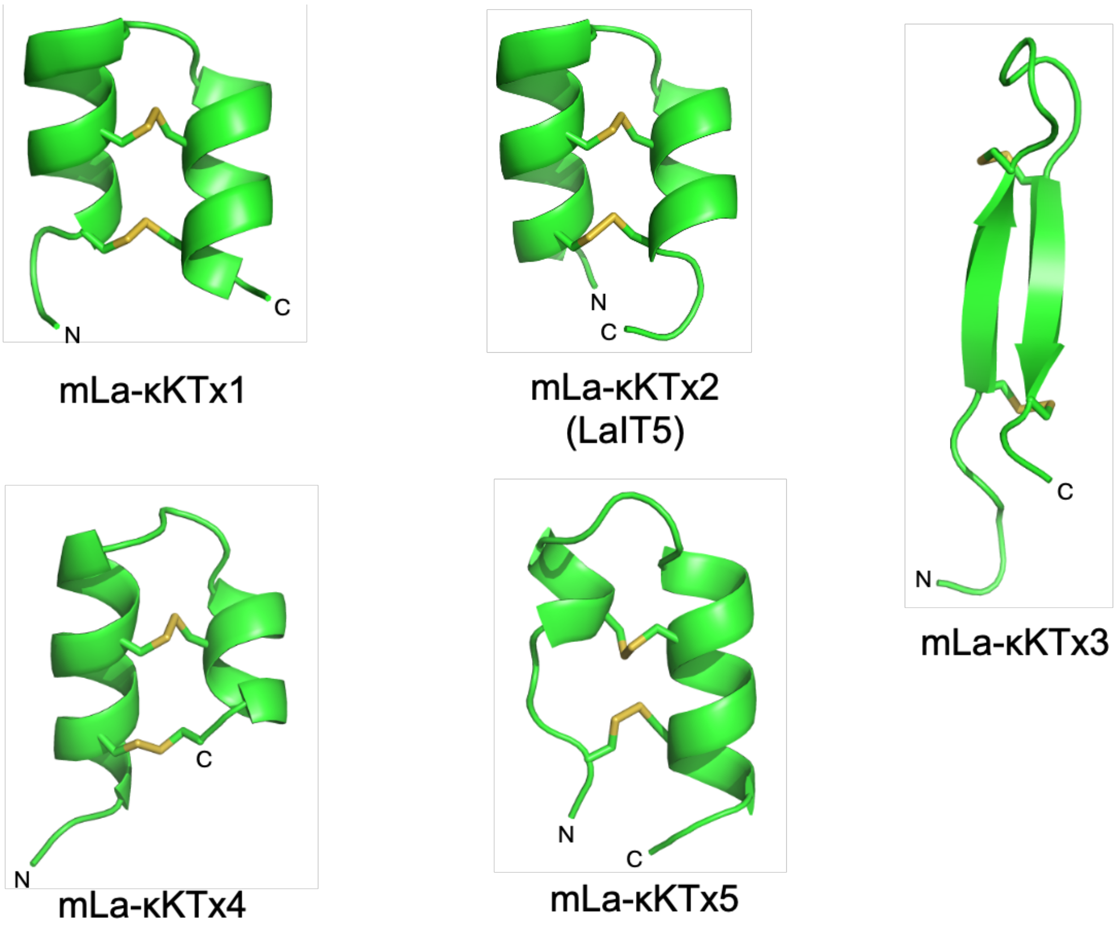

Figure S5 Prediction of 3D structures of the κ-KTx-like peptides the *L. australasiae* venom.

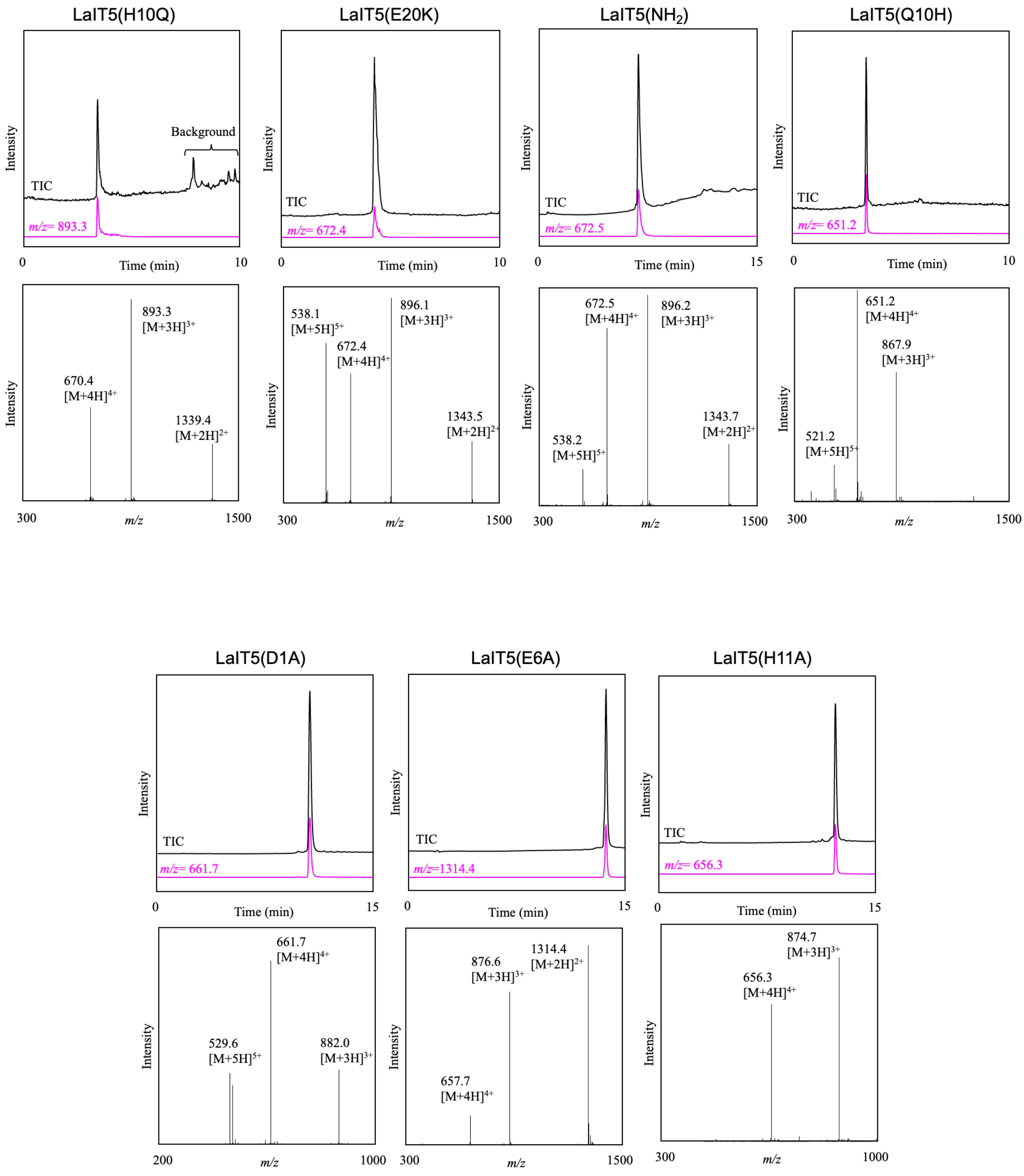
LaIT5(H10Q)
Intensity
Background
TIC
m/z= 893.3
0
Time (min)
10
893.3
[M+3H]3+
670.4
[M+4H]4+
1339.4
[M+2H]2+
300
m/z
1500
LaIT5(E20K)
TIC
m/z= 672.4
0
Time (min)
10
538.1
[M+5H]5+
672.4
[M+4H]4+
896.1
[M+3H]3+
1343.5
[M+2H]2+
300
m/z
1500
LaIT5(NH2)
TIC
m/z= 672.5
0
Time (min)
15
672.5
[M+4H]4+
896.2
[M+3H]3+
538.2
[M+5H]5+
1343.7
[M+2H]2+
300
m/z
1500
LaIT5(Q10H)
TIC
m/z= 651.2
0
Time (min)
10
651.2
[M+4H]4+
867.9
[M+3H]3+
521.2
[M+5H]5+
300
m/z
1500
LaIT5(D1A)
TIC
m/z= 661.7
0
Time (min)
15
661.7
[M+4H]4+
529.6
[M+5H]5+
882.0
[M+3H]3+
200
m/z
1000
LaIT5(E6A)
TIC
m/z=1314.4
0
Time (min)
15
876.6
[M+3H]3+
1314.4
[M+2H]2+
657.7
[M+4H]4+
300
m/z
1500
LaIT5(H11A)
TIC
m/z= 656.3
0
Time (min)
15
874.7
[M+3H]3+
656.3
[M+4H]4+
300
m/z
1000

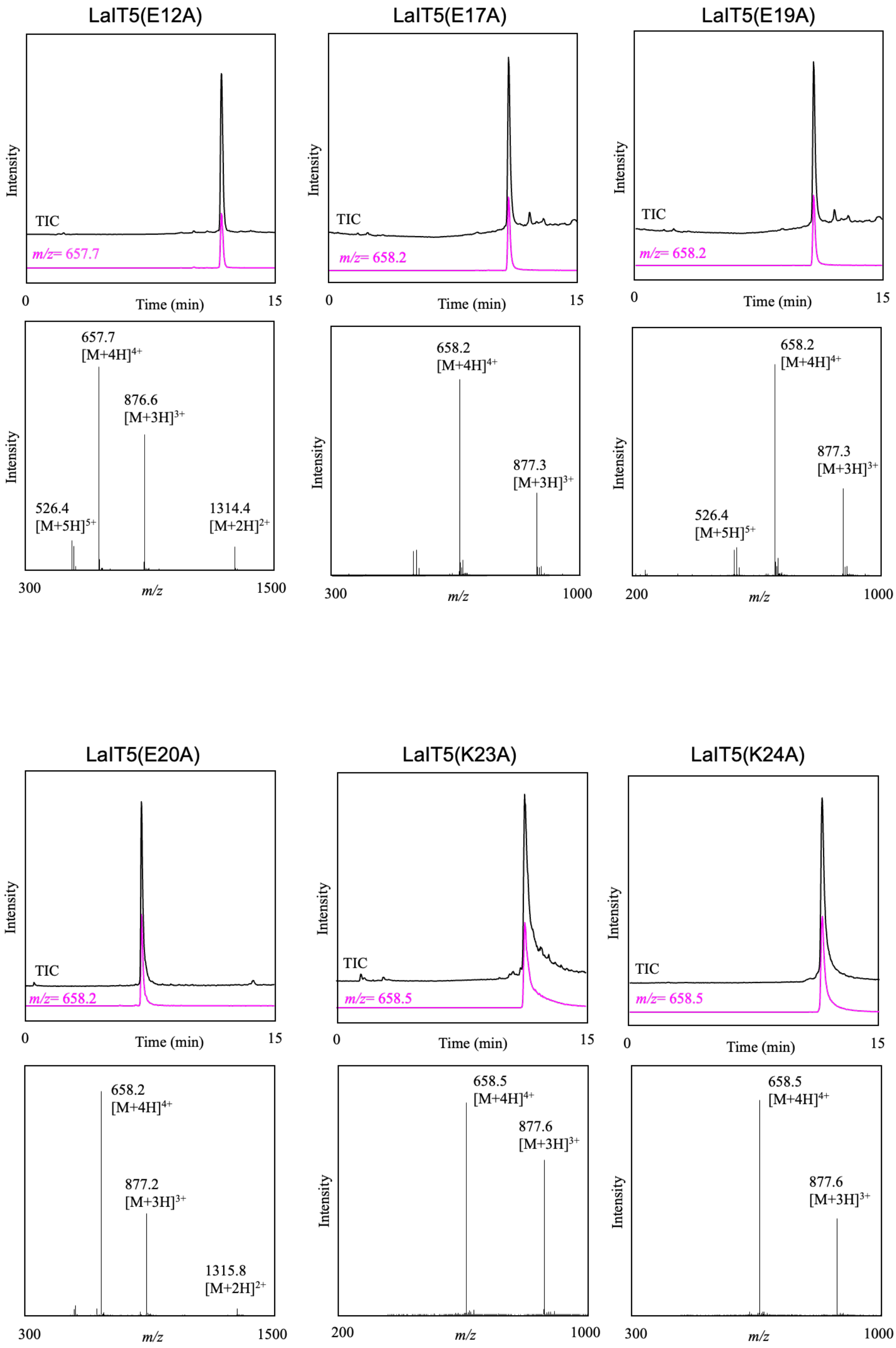

Figure S6 LC/MS analysis of the synthesized LaIT5 analogs after HPLC purification.
